# Understanding the Omicron Variant Impact in Healthcare Workers: Insights from the Prospective COVID-19 Post-Immunization Serological Cohort in Munich (KoCo-Impf) on Risk Factors for Breakthrough and Reinfections

**DOI:** 10.3390/v16101556

**Published:** 2024-09-30

**Authors:** Christian Janke, Raquel Rubio-Acero, Maximilian Weigert, Christina Reinkemeyer, Yeganeh Khazaei, Lisa Kleinlein, Ronan Le Gleut, Katja Radon, Marlene Hannes, Francesco Picasso, Anne Elisabeth Lucke, Michael Plank, Irene Charlotte Kotta, Ivana Paunovic, Ana Zhelyazkova, Ivan Noreña, Simon Winter, Michael Hoelscher, Andreas Wieser, Helmut Küchenhoff, Noemi Castelletti, on behalf of the ORCHESTRA Working Group

**Affiliations:** 1 Institute of Infectious Diseases and Tropical Medicine, LMU University Hospital, LMU Munich, 80802 Munich, Germany; christian.janke@lrz.uni-muenchen.de (C.J.); raquel.rubio@med.uni-muenchen.de (R.R.-A.);; 2 Statistical Consulting Unit StaBLab, Department of Statistics, LMU Munich, 80539 Munich, Germany; 3 Munich Center for Machine Learning (MCML), 80539 Munich, Germany; 4 Fraunhofer Institute for Translational Medicine and Pharmacology ITMP, Immunology, Infection and Pandemic Research, 80799 Munich, Germany; 5 Institute of Computational Biology, Helmholtz Munich, 85764 Neuherberg, Germany; 6 Core Facility Statistical Consulting, Helmholtz Munich, 85764 Neuherberg, Germany; 7 Institute and Outpatient Clinic for Occupational, Social and Environmental Medicine, LMU University Hospital, LMU Munich, 80336 Munich, Germany; 8 Center for International Health (CIH), LMU University Hospital, LMU Munich, 80336 Munich, Germany; 9 Comprehensive Pneumology Center (CPC) Munich, German Center for Lung Research (DZL), 89337 Munich, Germany; 10 Unit Global Health, Helmholtz Zentrum München, German Research Centre for Environmental Health (HMGU), 85764 Neuherberg, Germany; 11 Institut für Notfallmedizin und Medizinmanagement (INM), LMU Klinikum, LMU München, 80336 Munich, Germany; 12 German Center for Infection Research (DZIF), Partner Site Munich, 80802 Munich, Germany; 13 Max Von Pettenkofer Institute, Faculty of Medicine, LMU Munich, 80336 Munich, Germany; 14 Institute of Radiation Medicine, Helmholtz Zentrum München, 85764 Neuherberg, Germany

**Keywords:** COVID-19, SARS-CoV-2, health care workers, vaccination, immunologic response, antibodies, seroprevalence, breakthrough infections, reinfections, ORCHESTRA

## Abstract

This study analyzes immune responses to SARS-CoV-2 vaccination and infection, including asymptomatic cases, focusing on infection risks during the Omicron wave, particularly among high-risk healthcare workers. In the KoCo-Impf study, we monitored 6088 vaccinated participants in Munich aged 18 and above. From 13 May to 31 July 2022, 2351 participants were follow-uped. Logistic regression models evaluated primary, secondary, and breakthrough infections (BTIs). Roche Elecsys^®^ Anti-SARS-CoV-2 assays detected prior infections (via anti-Nucleocapsid antibodies) and assessed vaccination/infection impact (via anti-Spike antibodies) using dried blood spots. Our findings revealed an anti-Nucleocapsid seroprevalence of 44.1%. BTIs occurred in 38.8% of participants, with reinfections in 48.0%. Follow-up participation was inversely associated with current smoking and non-vaccination, while significantly increasing with age and receipt of three vaccine doses. Larger household sizes and younger age increased infection risks, whereas multiple vaccinations and older age reduced them. Household size and specific institutional subgroups were risk factors for BTIs. The anti-Nucleocapsid value prior to the second infection was significantly associated with reinfection risk. Institutional subgroups influenced all models, underscoring the importance of tailored outbreak responses. The KoCo-Impf study underscores the importance of vaccination, demographic factors, and institutional settings in understanding SARS-CoV-2 infection risks during the Omicron wave.

## 1. Introduction

The initial documentation of the emergence of COVID-19, attributed to the severe acute respiratory syndrome Coronavirus 2 (SARS-CoV-2), dates back to 31 December 2019, in Wuhan, located in the Hubei province of China [1]. Recognizing the widespread impact, the World Health Organization (WHO) officially declared COVID-19 a pandemic on 11 March 2020, in response to a surge in cases exceeding 118,000 across 114 countries, resulting in 4291 fatalities [2]. Following this declaration, global outbreaks ensued, with an estimated 775 million confirmed cases and more than 7 million deaths reported as of July 2024 [3].

On 27 January 2020, the Ludwig-Maximilians-University (LMU) Hospital’s Institute of Infectious Diseases and Tropical Medicine diagnosed the first German COVID-19 patient. The crucial revelation of the transmissibility of SARS-CoV-2 by asymptomatic carriers was evidenced through the observed transmission patterns in this case [4]. However, as the fourth anniversary of this event transpired, the current infection risk faced by healthcare workers (HCWs) in close contact with SARS-CoV-2-infected patients, as well as the broader SARS-CoV-2 infection risk among HCWs in general, remains inadequately defined, since most data stems from the pre-omicron phase of the pandemic [5,6,7,8]. During the initial pandemic waves, HCWs, grappling with an unfamiliar threat and experiencing acute shortages of critical personal protective equipment (PPE), were among the first victims of nosocomial infection chains [9,10,11,12]. Reports from this period indicated that, among other factors, PPE use [13], vaccination status, and exposure location were relevant determinants of risk [14,15,16,17]. However, even then, findings underscored the substantial influence of factors such as male sex and Eastern European nationality, suggesting that factors beyond institutional exposure patterns might play a critical role in overall infection risk [18].

In May 2021, the longitudinal cohort named KoCo-Impf (Prospective COVID-19 post-immunization Serological Cohort in Munich—Determination of immune response in vaccinated subjects) was established at the Institute of Infectious Diseases and Tropical Medicine. It predominantly comprises HCWs with high contact risk with the SARS-CoV-2 virus but also non-HCWs categorized as members of the general population [14]. The primary focus of the cohort was to identify the risk factors for infection in HCWs and compare them with the general population of the same cohort. Additionally, KoCo-Impf runs alongside KoCo19, a large longitudinal cohort that focuses on a representative subset of the Munich general population [19].

As pandemic waves progressed, new variants emerged, and vaccine boosters were introduced, the situation became increasingly complex. Therefore, in May 2022 we conducted a follow-up analysis focusing on the impact of the omicron variant.

The Omicron variant, first identified in Botswana and South Africa in November 2021, is the fifth variant to be classified as a Variant of Concern by the WHO. The initial B.1.1.529 lineage has diverged into multiple sub-lineages, with BA.1 initially prevalent but quickly overtaken by BA.2, becoming globally dominant. Omicron’s numerous mutations in the spike protein enable it to evade immunity from both prior infections and vaccinations, leading to a higher susceptibility to reinfections and breakthrough infections [20,21,22]. This ability to evade immune defenses has driven a rapid surge in global COVID-19 cases despite widespread vaccination efforts. Additionally, Omicron’s higher transmissibility but generally less severe course has resulted in more silent and undetected infections [23].

This underscores the strength of our strategy utilizing the detection of antibodies generated after SARS-CoV-2 infection and/or vaccination compared to other methods. By detecting anti-Nucleocapsid (anti-N) antibodies, we can identify undergone natural infections (or vaccinations with nucleocapsid-containing vaccines not commonly used in Europe), while with the anti-Spike (anti-S) antibodies, we can identify both natural infections and vaccinations [24,25].

In this analysis, we present the follow-up data of the KoCo-Impf cohort. Our aim is to describe, determine, and conceptualize the SARS-CoV-2 infection risk for HCWs during the first Omicron wave from May 2022 to July 2022. This study tracks a large cohort of mainly HCWs from various institutions across the greater Munich area, building upon previously reported baseline risk patterns [14]. We present differences between the risk factors of variants before and after Omicron. We explore breakthrough infections and risk factors for reinfection. Additionally, we address why risk assessment for this key population is challenging, investigating why scientific evidence remains limited and, at times, contradictory. 

## 2. Materials and Methods

### 2.1. The Follow-Up Logistics for the KoCo-Impf

**Figure 1:**
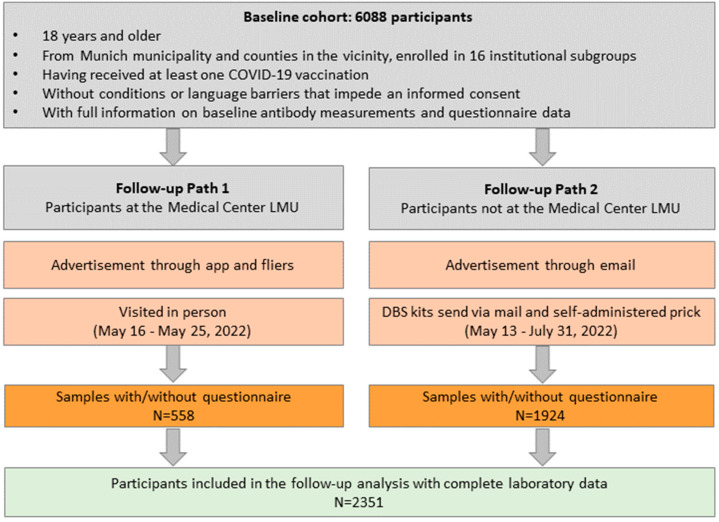
Recruitment paths and criteria for inclusion into the follow-up analysis. Gray boxes: institutional subgroups. Orange boxes: information on advertisement modalities for reaching to participants; modalities of the acquisition of questionnaire data, and capillary blood samples. Green Box: Inclusion criteria for follow-up analysis.

As previously outlined [14], there were differences in the management of participants between the HCWs at the Medical Center of the LMU and the remainder of the cohort (including the participants belonging to the general population). This organizational distinction also extended to the follow-up process, as delineated and compared in Figure 1. For the HCWs at the LMU Medical Center, follow-up information was disseminated through an app and distribution of fliers. Conversely, all other participants were contacted via email. The sampling methods varied further: HCWs of the LMU Medical Center underwent in-person visits from 16 May 2022 to 25 May 2022, during which capillary blood samples (DBS) were obtained on-site by trained personnel. In contrast, all other participants received the DBS kits via mail and self-administered the pricks. Sample returns occurred between 13 May 2022 and 31 July 2022. This standardized protocol has been consistently implemented across all follow-ups of KoCo19 [19]. 

### 2.2. Specimen Collection and Laboratory Analyses

The method of specimen collection transitioned from comprehensive in-person DBS sampling conducted by trained personnel at baseline to partial DBS self-administered pricks during follow-up [14]. For a more comprehensive understanding of the DBS analysis, please refer to [26,27]. The laboratory analysis method remained consistent between baseline and follow-up assessments. Two assays were employed: Ro-RBD-Ig for detecting antibodies post-infection and vaccination, and Ro-N-Ig for detecting antibodies post-infection only. The combination of both assays allows us to distinguish between infection and vaccination. Ro-N-Ig confirms a prior infection but does not provide the exact infection date. The DBS-seropositivity cut-off for Ro-RBD-Ig is 0.115 COI (cut-off index), while for Ro-N-Ig it is 0.105 COI. Both assays have been validated to ensure no cross-reactivity with viral infections occurring prior to the COVID-19 era, as confirmed by the analysis of blood samples from donors preceding the emergence of COVID-19 [24,25].

### 2.3. Data and Statistical Analysis

The baseline questionnaire data was used to identify risk factors for infection. Therefore, the data descriptions and variable definitions match those in the baseline manuscript [14]. However, the variable “cumulative cases” (the total number of infections up to a given point; see [14] for details) was not included in the models because the follow-up period was similar for the entire cohort, ensuring comparable “time under risk” between baseline and follow-up. The baseline variable “contact with positives” was also excluded as it was outdated for new infections. For individuals recruited on the day of vaccination, their vaccination status prior to recruitment was considered (e.g., non-vaccinated if it was their first shot, vaccinated once if it was their second shot, etc.). For the variable vaccination scheme, the category “1 vaccination” was used as the reference, unlike the baseline analysis where “not vaccinated” was considered the reference category. This change is because, for the follow-up analysis, all “not vaccinated” participants belong to the “general population” institutional subgroup. Analyses separating the general population from other institutional subgroups were conducted but showed no significant differences. Therefore, all the models presented here include all institutional subgroups, but interpretation requires careful attention.

Additionally, we focus on infections that occur after the completion of the vaccination regimen, specifically breakthrough infections, and reinfection (double infections). Reinfections were identified in participants who tested anti-N positive at baseline and showed a higher anti-N value at follow-up. Since anti-N indicates a prior infection and it is not induced by vaccinations used in Germany, the rise in anti-N between assessments suggests a second infection occurred between baseline and follow-up. To ensure accuracy, we excluded cases where the difference in values could be attributed to the inherent variability of the measurement. To determine this threshold, we compared two measurements of the same sample, calculated the difference in their log10 values, and determined the Standard Deviation (SD), which was 0.1305 COI. Any differences between baseline and follow-up measurements smaller than 2*SD were excluded from the reinfection definition.

The manuscript encompasses five distinct multivariable logistic regression models. One model evaluates the non-responder mechanism, while the other four assess the risk of different types of infection (anti-N seropositivity). Odds ratios (OR), 95% confidence intervals (CI), and *p*-values were calculated. Categorical variables are described with frequencies and percentages.

Model 1 describes the risk factor analysis for participants who contracted the SARS-CoV-2 virus at any time during surveillance (anti-N positive at baseline and/or follow-up vs. anti-N ever negatives). This approach is based on the assumption that risk factors for infection remain constant over time. Consequently, the number of infections increases over time, irrespective of when the infection occurred.

Model 2 focuses solely on new anti-N positives at follow-up vs. anti-N ever negatives, excluding positives at baseline. This approach allows for the examination of risk factors specific to Omicron infection.

Model 3 addresses breakthrough infections vs. anti-N ever negatives, where participants of both groups had at least two vaccinations at baseline (select participants with at least two vaccinations at baseline and exclude anti-N positives at baseline. Anti-N positives at follow-up vs. anti-N ever negatives). The analysis aims to understand the reasons for infection despite complete vaccination coverage. The baseline anti-S level is taken into account to assess potential levels of protection.

Model 4 delves into reinfection and compares individuals who experienced two infections (an increase in anti-N levels at follow-up compared to baseline) vs. subjects with only one infection (anti-N positive only at baseline with no increase in anti-N level at follow-up). The levels of anti-S and anti-N prior to reinfection (i.e., at baseline) are taken into account to assess potential levels of protection. Due to the sparse distribution of anti-N values above a COI value of 4, the nine values exceeding 4 were capped at 4.

The non-response mechanism over the follow-up was studied using a logistic regression coding with 1 for the participants that could be included in the analysis and 0 for the non-responders.

Missing data in the covariates for all five models were addressed through multiple imputations with m = 5 iterations. The response variables were also included in the imputation process to ensure unbiased regression coefficients [28]. Rubin’s rules were used to compute the total variance of coefficient estimates over the repeated analyses [29]. Model evaluation was carried out using the Area Under the Receiver Operating Characteristic Curve (AUC) value obtained from ten-fold cross-validation. 

All statistical analyses and visualization were performed using the R software (version 4.4.1, R Development Core Team, 2021). The models were estimated using the R package mgcv [30] and the visualization was done using the package APCtools [31].

## 3. Results

To exclude that the different engagement methods may have led to intrinsic behavioral differences, which could, in turn, influence the point estimates in the models, we reran all the models also excluding the general population and the LMU Medical Center. No significant differences in point estimates were observed with either exclusion. Therefore, only comprehensive models are presented here.

### 3.1. Non-Responder Mechanism and Follow-Up Cohort Description

**Figure 2:**
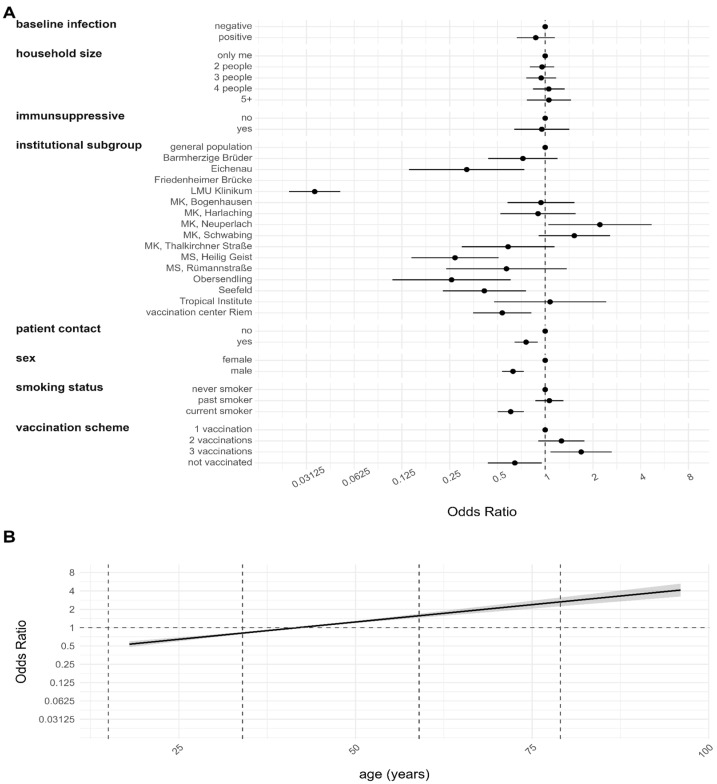
Non-response mechanism at the follow-up using multiple imputation. Results are based on a logistic regression model and are given as ORs with a 95% CI. The outcome was coded with 1 for participants that could be included in the follow-up analysis and 0 for non-responders. The obtained value of the model evaluation unison pooled AUC was 0.8595. (**A**) Estimates for categorical variables. (**B**) Estimates for continuous variables with 95% CI represented by the gray shaded region.

The non-responder analysis results are depicted in Figure 2. Estimates for the institutional subgroup Friedenheimer Brücke were omitted from the plot since all members participated in the follow-up. However, the participant information of this institutional subgroup was retained for all other variables.

Compared to the general population, institutional subgroups Eichenau, LMU Medical Center (LMU Klinikum), MS Heilig Geist, Obersendling, Seefeld, and the vaccination center Riem were less likely to participate in the follow-up. HCWs with patient contact and male participants were less inclined to participate. Current smokers were less inclined to participate than never smokers. Additionally, unvaccinated individuals were less likely to participate than vaccinated individuals, with vaccinated twice showing no significant difference to only once vaccinated. Participation also statistically significantly increased with increasing age. Interestingly, only the institutional subgroup MK Neuperlach showed significantly higher participation compared to the general population. Covariates such as anti-N sero-positivity at baseline, household size, and intake of immunosuppressive drugs showed either no association or non-significant ones with non-response behavior.

Table 1 describes the follow-up cohort, comprising 2351 participants, focusing also on breakthrough infections and reinfections. Within this diverse cohort, 44.1% (1036/2351) tested anti-N positive. Breakthrough infections were observed in 38.8% (695/1793) of participants having at least two vaccinations and being anti-N negative at baseline. In total, 48.0% (84/175) of the participants being anti-N positive at baseline experienced reinfections. Looking at the demographic patterns, among the participants, 1740 females and 611 males participated, with 42.6% (741/1740) and 48.3% (295/611), respectively, testing anti-N positive. Institutional subgroups displayed variations, showcasing different anti-N positivity rates. Participants with patient contact exhibited a 44.9% (544/1211) positivity rate, while those without contact showed only 40.4% (308/762) sero-positivity. Smoking habits also played a role, with 44.9% (735/1636) of never smokers testing positive, compared to 39.7% (136/343) among current smokers and 44.7% (164/367) among past smokers. All unvaccinated participants belong to the general population subgroup and a significant portion (75.7%, 1779/2351) of the cohort had already received two vaccinations at baseline, with 41.8% (744/1779) testing anti-N positive. The group with intake of immunosuppressive drugs showed a 41.0% (34/83) positivity rate. Household dynamics indicated that larger households (four people or more) exhibited an increased anti-N positivity rate of 53.2% (249/468) or higher, while one-person households showed a 39.3% (262/667) positivity rate.

### 3.2. Development of the Antibodies over Time: Group Characterization and Vanishing Effect of Vaccination

**Figure 3:**
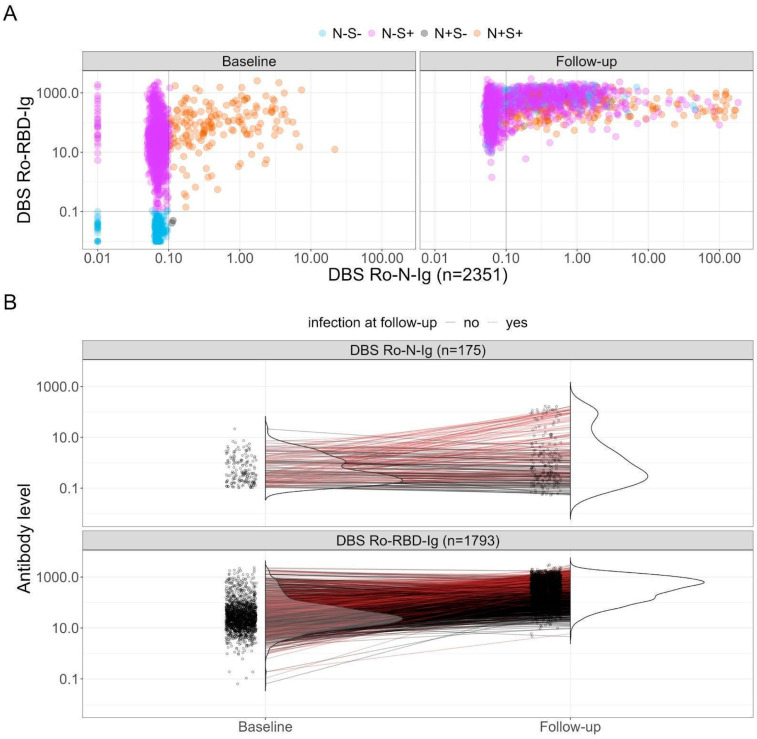
Analysis of anti-N and anti-S antibodies over time at baseline and follow-up. (**A**) Scatterplot displaying raw values of anti-N and anti-S antibodies (*n* = 2351). The Ro-N-Ig measurement is abbreviated with “N”, while Ro-RBD-Ig is represented as “S”. Positivity is indicated with “+”, negativity is denoted with “-”. The color code is determined by the subjects’ status at baseline. (**B**) Top: Assessment of individuals who were anti-N positive at baseline, with a focus on the identification of reinfections (*n* = 84, depicted in red). Bottom: Examination of participants with at least two vaccinations and were anti-N negative but anti-S positive at baseline (*n* = 1793). Breakthrough infections are identified with a positive anti-N result at follow-up and are denoted in red (*n* = 695).

Examining the progression of SARS-CoV-2-related antibodies over time provides crucial insights into the cohort’s evolution. This assessment involves plotting anti-N and anti-S antibodies on the x- and y-axes, respectively (see Figure 3A). The left side represents the baseline status, while the right side represents the follow-up status. The color scheme corresponds to the baseline result, with naive participants in blue (negative in both), solely vaccinated individuals in pink (anti-S positive but anti-N negative), those vaccinated and/or infected in orange (positive in both), and in gray individuals infected but with anti-S non-responder or late-responder status after infection, or a false positive value for anti-N. Observing the follow-up values, we notice a shift; no participants remain naive (quadrant bottom left of the follow-up plot is empty), and there is a shift to the right in the anti-N values of the infected and vaccinated group (orange dots), signifying potential reinfections. Additionally, we observe an increase in the number of solely vaccinated participants who became infected (shift to the right of pink dots), indicating breakthrough infections.

Reinfections were identified by comparing positive anti-N values of participants at baseline and the values at follow-up (see Figure 3B top). For some participants, anti-N levels decreased, indicating a natural decline in antibody levels (denoted in black). Conversely, for others, an increase signaled a reinfection (denoted in red). In this case, the baseline values of participants who experienced reinfection varied across the entire range, suggesting that Omicron reinfections may not be dependent on this variable. A similar pattern can be found by looking at the anti-S baseline values. However, it is advisable to include these variables in subsequent models to further elucidate their potential role in protection.

To further analyze breakthrough infections, we focused on at least double vaccinated participants with a negative anti-N value at baseline and examined the change in anti-S levels in the follow-up (see Figure 3B bottom). Participants showing a positive anti-N at follow-up are marked in red. Participants showed a clear anti-S increase, which could be attributed to an additional vaccination or an infection. Also, for breakthrough infections, the baseline values varied across the entire anti-S range.

### 3.3. Risk Factor Analysis for the Anti-N Sero-Positivity during Different Observation Periods

**Figure 4:**
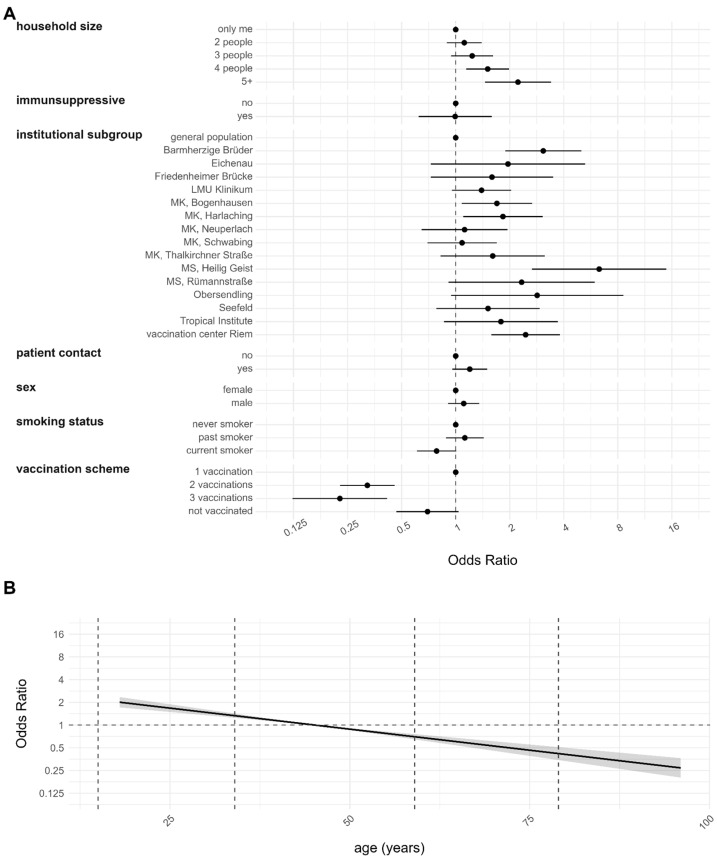
Risk factor analysis for infection at any time point in the study period (*n* = 2351, Model 1). A person with a prior infection was identified as being anti-N positive either at baseline, follow-up, or both (ever positive definition). Findings are derived from multiple imputations. The obtained value of the model evaluation unison pooled AUC was 0.6485. (**A**) Estimates for categorical variables. (**B**) Estimates for continuous variables with 95% CI represented by the gray shaded region.

The results of the risk factor analysis for individuals who tested sero-positive in anti-N either at baseline and/or follow-up (referred to as ever positives, Model 1) are presented in Figure 4. The analysis revealed that individuals living in households with more than four residents were more susceptible to infection compared to those living alone. Similar tendencies were observed for household sizes of two and three, although these were not statistically significant. Among the institutional subgroups, only five centers (Barmherzige Brüder, MK Bogenhausen, MK Harlaching, MS Heilig Geist and vaccination center Riem) exhibited an increased risk for infection compared to the general population.

Participants who received more than one vaccination were less likely to contract SARS-CoV-2 compared to those who received only a single vaccination. No statistically significant difference in infection risk was observed between participants who were not vaccinated and those vaccinated only once. Younger participants demonstrated an increased risk, whereas individuals older than 50 years exhibited a lower risk of infection. Other factors such as the intake of immunosuppressive drugs, patient contact, sex, and smoking status did not show a significant influence on the risk of infection.

When exclusively examining new anti-N-seropositive cases during follow-up, with a specific focus on Omicron infections, the identified risk factors remained unchanged (Supplemental Figure S1, Model 2, *n* = 2176). However, when considering the institutional subgroup variable, only the institutions MS Heilig Geist and vaccination center Riem remained statistically significant. Although the effects related to vaccination remained statistically significant, they were observed to be less pronounced.

### 3.4. Risk Factor Analyses for Infection after Complete Vaccination and Reinfection

**Figure 5:**
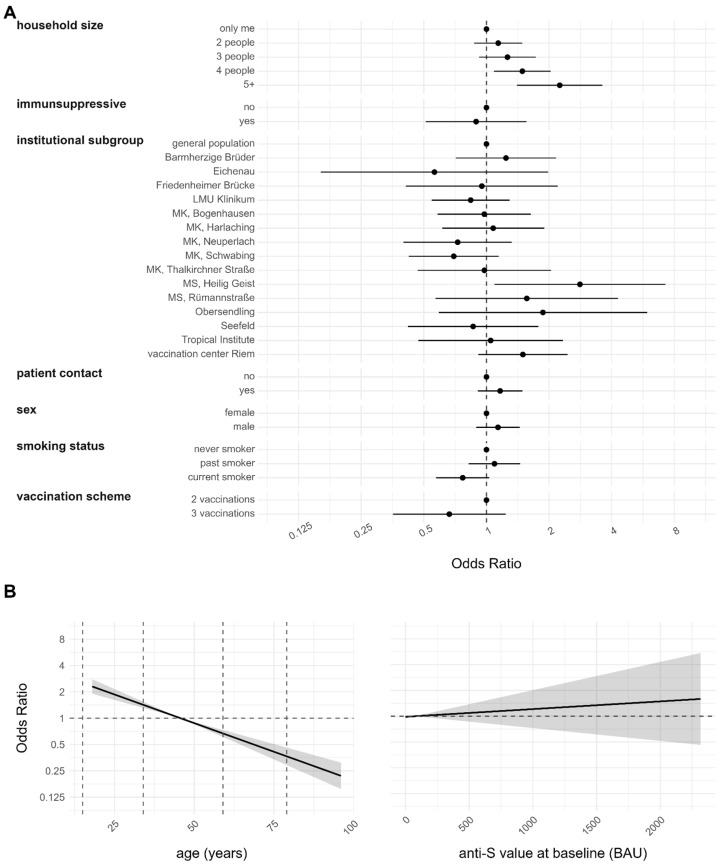
Risk factor analysis for breakthrough infection (*n* = 1793, Model 3). A person with a breakthrough infection was identified as having at least two vaccinations at baseline and being anti-N positive only at follow-up (anti-N negative at baseline but positive at follow-up). AS comparison only anti-N ever negatives with at least two vaccinations at baseline were selected. Findings are derived from multiple imputations. The obtained value of the model evaluation unison pooled AUC was 0.6302. (**A**) Estimates for categorical variables. (**B**) Estimates for continuous variables with 95% CI represented by the gray shaded region.

Model 3 aims at identifying the risk factors for infection among individuals who have completed their vaccination regimen, comparing those who have been double or more vaccinated and subsequently infected with those who have received only vaccination and were not subsequently infected (*n* = 1793). The findings are illustrated in Figure 5. The only covariates that indicated an elevated risk for breakthrough infection were household size, institution subgroup and age above 50 years.

Consistent with our previous analyses, only households with four or more occupants exhibited a significantly increased risk compared to individuals living alone. Similar trends were observed for households with two and three members, although these were not statistically significant. The institutional subgroup MS Heilig Geist exhibited a higher risk compared to the general population. Interestingly, having received three vaccinations did not show any significant difference compared to having received only two. The anti-S value at baseline was included in the analysis to study a potential protective effect but the variable did not show any statistical significance.

The risk of reinfection, comparing individuals infected only once to those with double infections, was analyzed in Model 4 (*n* = 175). The variable ‘institutional subgroup’ was excluded from the analysis due to insufficient individuals categorized for each institution. The model’s findings are depicted in Supplemental Figure S2. Among the variables examined, only the anti-N value prior to the second infection exhibited a statistically significant association with reinfection. Specifically, a lower baseline value indicated a protective effect compared to a higher one.

## 4. Discussion

In this investigation, we examine the factors associated with COVID-19 infections within a study group inclusive of both, the general population and HCWs, who encounter elevated exposure risks to the SARS-CoV-2 virus. We employed capillary blood specimens to ascertain the presence of SARS-CoV-2 antibodies, serving as indicators of prior infections encompassing symptomatic and asymptomatic instances, alongside vaccination records. As a follow-up of the cohort presented previously [14], our focus shifts to discerning differences in risk factors among virus variants, instances of infection post-completion of the vaccination regimen, and factors contributing to reinfection.

The variable “institutional subgroup” has proven to be highly significant already in the baseline analyses, emerging as the most influential factor [14]. This significance persists in this subsequent follow-up analysis of all the models. This demonstrates the considerable variation in institutional structures and, consequently, in the rates of new infections across different institutions. Similarly, protective measures should be tailored to the specific contexts of each institution, moving beyond generalized approaches such as the use of PPE. Rather, a nuanced understanding of infection transmission dynamics within each institution is imperative. In addition, the variance in SARS-CoV-2 transmission risks observed among distinct institutions in our study may not solely be indicative of differences in risk-associated behaviors, procedural implementations, or adherence to PPE guidelines. Such disparities may be rooted in the fundamental characteristics of SARS-CoV-2 dissemination, which is typified as a series of hyperlocal events [32]. Both interpretations highlight the intricacies of transmission dynamics, proposing that a confluence of broader contextual factors alongside stochastic elements substantially influences the likelihood of institutional SARS-CoV-2 outbreaks.

Since the institutional subgroup was one of the strongest variables in the analyses, all models were also run separately for the general population only and all other institutional subgroups were combined to assess the impact of this variable on the other observed effects. No relevant differences were found, except in Model 1, where patient contact was significant only for the general population (OR for patient contact: (i) general population 2.69 [1.07–6.76], (ii) all other institutions 1.20 [0.96–1.50],). This might indicate that HCWs are better able to protect themselves from possible infections compared to individuals from the general population not being classified as HCWs but carrying out activities involving patients. For all the risk factor analyses presented here, generalized linear mixed effects models (GLMM) with institutional subgroup as random intercept would also have been appropriate, as this accounts for similar behavior among individuals from the same institution. However, it was crucial to include the coefficients of the different institutional subgroups to allow for direct comparison between hospitals. The possible power loss was manageable, and the coefficients remain interpretable. The risk profiles of institutional subgroups varied across baseline, follow-up, and Omicron-only cases, reflecting fluctuations in infection rates relative to the general population over time. Given the ongoing nature of the pandemic, this aspect warrants careful consideration and interpretation alongside the evolving waves and timelines of the pandemic. A time-to-event analysis, such as Cox regression, would not adequately address this feature and is therefore unsuitable for this analysis. This analysis can also serve as a sensitivity assessment for case numbers. 

Numerous publications have undertaken analyses of infection risks among HCWs. For instance, part of our data has contributed to the examination of determinants of anti-S immune response at 6, 9, and 12 months post-COVID-19 vaccination within a multicentric European cohort as part of the ORCHESTRA project [33,34,35]. While relative risks were adjusted for country and, in some cases, institutional subgroup, the robustness of these findings may still be influenced by the strength of the institutional subgroup effect, which might just be a proxy for local outbreaks. Consequently, analyses involving multicentric cohorts offer expanded and arguably more representative population samples but may yield less reliable results compared to those from single-center analyses. Similarly, the analysis of larger hospitals is contingent upon the specific departments to which HCWs are assigned. Therefore, comprehensive investigations into infection transmission mechanisms across different departments and institutions are warranted.

Examining the non-responder mechanism, it was observed that non-vaccinated and younger participants demonstrated less inclination to engage in the follow-up analysis. This phenomenon could stem from the perception that these groups do not perceive themselves to be at risk and therefore lack interest in monitoring new infection rates. Conversely, it may also be the case that these individuals, being aware of their heightened exposure to potential infections, already consider themselves at elevated risk and hence do not require further quantification from the study. Previous research has already reported lower non-responder rates of younger healthcare workers [36]. Another possibility could be that at the vaccination center Riem, we recruited younger participants who had recently been vaccinated. It is plausible that these young participants had moved out of Munich and therefore could not participate in the follow-up.

The primary focus of the KoCo-Impf cohort is to identify the risk factors for infection in HCWs and compare them also with the general population. In addition to the general population of KoCo-Impf itself, for this comparison, recruitment occurred concurrently with the third and fourth follow-up of KoCo19 in Munich, a prospective and Munich-representative COVID-19 cohort, although comparing the two cohorts poses substantial challenges [19]. Notably, the variables of sex and age exhibited similar patterns of missing data compared to the KoCo19 cohort [19,36], indicating that despite the focus on HCWs, this study can provide insights applicable to the broader population. Intriguingly, prior infection status at recruitment did not exhibit statistical significance in terms of missing data, a contrast to findings in KoCo19 [19,36]. This discrepancy may be attributed to several factors. Firstly, the level of interest in infection dynamics might differ between the general population and HCWs, with the latter, perceiving a heightened risk, displaying sustained interest even after a previous infection. Secondly, it could be influenced by the different timing of follow-up assessments. During the KoCo-Impf follow-up, the emergence of the Omicron variant and the understanding that previous infections might not confer immunity against subsequent infections became increasingly pertinent. Consequently, risk perceptions evolved over time, aligning with findings from other studies [37,38,39]. Although comparing the two cohorts posed challenges and required careful evaluation, it was confirmed in both cohorts that HCWs had a higher risk of infection. Sex, age, household size, and intake of immune-suppressing drugs were not found to be significant risk factors for infection in either cohort, but being a current smoker was [14,19]. The lower number of detected cases in the KoCo-Impf, however, indicates a more complex scenario. Disparities in vaccination timing, behavioral adaptations, and methodological challenges in comparing the representative KoCo19 with the convenience sample of HCWs could potentially influence, or even bias, the assessment of exposure and infection risk.

In the follow-up analysis, risk factors for infection—including household size, current smoking status, and institutional subgroup—showed changes compared to baseline (see Figures 2 and 4 of [14]). Household sizes of four or more exhibited statistically significant increases in infection risk during the follow-up, a pattern not observed at baseline. This shift may be attributed to the predominance of Omicron infections, which are more closely linked to contact intensity [40]. Larger household sizes correspond to higher probabilities of virus exposure, consistent with findings that the Omicron variant is considerably more contagious than previous variants [20,21,22,40]. During lockdown, the HCWs had possibly the most external contacts due to their job. With the lifting of lockdowns, the impact of having more individuals in the households became evident. The confirmation that this effect primarily stems from Omicron infections, rather than merely a larger sample size, is supported by risk factor analysis focusing solely on Omicron cases (see Supplemental Figure S1). Households with smaller household sizes showed similar effects but were not significant. This might just be due to a too-small sample size. Interestingly, current smokers exhibited a lower risk of infection in the baseline analysis, a trend that persisted in the follow-up but did not reach statistical significance. This change may be attributable to fluctuations in sample size. However, this effect was previously discussed in the baseline paper [14] and has been observed in other independent cohorts [41,42,43,44,45] as well as in the RisCoin cohort [46]. The estimates for the institutional subgroups remained highly significant in the follow-up analysis, although their magnitude diminished (see Figure 2 of [14] compared to Figure 4). This may indicate a leveling of infection risk over time between the general population and the other institutions. During the Omicron period, only two institutional subgroups remained statistically significantly different from the general population (see Figure 4 and Supplemental Figure S1). This suggests that the risk of institutional subgroups was more similar to the general population in the Omicron period than in the pre-Omicron period.

Another distinctive feature associated with the Omicron variant is its impact on the SARS-CoV-2 infection risk among HCWs compared to the general population. Previous studies [9,10,12], including our baseline analysis [14], highlighted a significantly elevated infection risk for HCWs, particularly those in patient-facing roles [46,47], during the first waves of the pandemic. However, in this follow-up analysis, an increased risk is not evident when analyzing Omicron infections exclusively (refer to Supplemental Figure S1). Factors such as enhanced personal infection protection practices in healthcare settings and the Omicron variant’s notably higher reproduction rate facilitating its widespread dissemination across traditionally low-risk environments may have led to an equalization of risk across populations. This phenomenon aligns with the outcomes of additional research [40,48], indicating a ‘socialization’ of infection risks at least since the emergence of the Delta variant. However, other studies still found a higher proportion of infected HCWs compared to the general population during the first Omicron wave [49]. Additionally, the Omicron infections result in a decreased hospitalization rate, leading to fewer infectious individuals in the hospitals. This inevitably reduces the difference in infection pressure between hospitals and the general community.

When comparing Omicron to non-Omicron infections, the most notable difference is observed in the vaccination status variable. The direction of effects remains consistent, with participants who received two or three vaccinations demonstrating a protective effect compared to those vaccinated only once. However, the magnitude of these effects notably decreases when examining Omicron infections. This reduction in effectiveness is attributed to the waning protection of vaccinations against Omicron variant infections [20,21,50]. 

For breakthrough infections, there has been no identified correlate of protection based on the anti-S baseline value. This observation does not necessarily indicate the absence of a protective threshold. Rather, it suggests that the value fluctuates depending on the viral load to which an individual is exposed relative to the contagiousness of the current SARS-CoV-2 variant. Exposure levels can vary significantly. It is conceivable that an individual with assumed low protection (characterized by a low anti-S level) may encounter a low viral load, thereby preventing infection as the immune system can intercept the infection before symptoms manifest. Conversely, it is possible that an individual considered with high protection (characterized by a high anti-S level) may encounter such a high viral load that protection is rendered ineffective. Although this scenario may result in non-significance in risk factor analysis, it underscores the presence of probably relevant biological meaningful values. The same argument can be brought with the neutralization capacity of the exposed subject, making the identification of a correlate of protection even more challenging.

Other studies have examined in vitro neutralization levels to identify correlates of protection, revealing a non-linear relationship. While this approach offers a possible solution to the issue, it was not feasible in our case due to the use of DBS sampling and a much larger sample size [51]. Similar to our analysis, other studies have investigated antibody responses, finding that higher anti-S levels were associated with a reduced risk of reinfection, while no association was found for anti-N levels. This discrepancy may be attributed to differences in sample composition, as all donors in those studies were vaccinated prior to sampling, potentially leading to a distinct antibody response [52].

In a comprehensive multicenter analysis of breakthrough infections [53], which incorporated an earlier subset of our data, significant correlations were observed between infection risk and the number of booster doses received. In our analysis, this was not the case. This divergent finding could be attributed to several factors: the impact of pre-Omicron variant infections, a shorter observational timeframe, considerable variability among study centers with notably high rates of breakthrough infections in Northern Italy, enhanced statistical robustness stemming from a larger sample size under investigation, and different approaches in case definition. 

Regarding reinfections, no demographic factor despite age exhibited a statistically significant association, suggesting that reinfection could potentially affect any individual or that the specific variable under scrutiny remains unknown. This observation may be attributed to the diminished protective effect of prior pre-Omicron infections against Omicron SARS-CoV-2 infections, a phenomenon documented in numerous studies following the emergence of this variant [54]. Nonetheless, the inconsistency in the definition of reinfection across the literature complicates direct comparisons. In our analysis, reinfections correlate with an increase in the anti-N baseline value. This may seem counterintuitive, as one might anticipate greater protection with higher antibody levels [55]. However, elevated anti-N values can also reflect the behavior of the participant. Higher values could indicate increased exposure to the virus through more frequent contacts. Alternatively, individuals exhibiting elevated anti-N values following a SARS-CoV-2 infection may represent a subset more susceptible to severe COVID-19 outcomes [56,57]. This subgroup could inherently possess risk factors not considered in this analysis predisposing them to infection initially. 

The cohort was recruited between June and December 2021, allowing participants a maximum of 22 months to contract the infection prior to recruitment. It is possible that some participants who were negative at baseline had been infected earlier but had reverted to seronegative status, thus excluding them from the reinfection analysis. However, a drop in seronegative status between study rounds can be ruled out, as discussed in Kroidl et al. [58].

The pattern of younger participants facing a heightened risk persisted across all facets of our analysis, encompassing the general infection risk (Model 1), the risk specific to Omicron (Model 2), and the risks associated with breakthrough infections and reinfections (Models 3 and 4). This observation is consistent with the results of other investigations [53]. However, some studies have identified a more nuanced relationship between age and these risks [50], while others have noted an elevated risk among older populations [59]. Notably, within our framework, the influence of age appears to be more behavioral than biological.

The analysis presented primarily focuses on (re)infections and breakthrough infections within the KoCo-Impf study cohort. We have demonstrated that the differences among institutional subgroups are a fundamental factor. The capacity to identify institutional disparities in our study was enabled by the strategic timing of follow-up evaluations, conducted at analogous time points across all 15 institutions. Such disparities are likely to be overlooked in studies that focus on single institutions or in meta-analyses that include follow-ups conducted at varying times. This highlights the critical importance of uniform temporal alignment in observational research to capture nuanced differences between institutions effectively. Operationally, this insight underscores the imperative for the development and implementation of localized outbreak management and rapid response mechanisms, tailored to the different needs of each institution in space and time. This approach should form the basis for better safeguarding this essential sector of our society.

Finally, if ‘every good regulator of a system must be a model of that system’ [60], our results imply that the outbreak management for HCWs in the era of the Omicron variant should extend the scope of strategies beyond the healthcare facilities. The healthcare environment was a primary risk factor at the pandemic’s outset. However, our findings indicate the importance of considering the wider environmental risks HCWs face within their personal households and social circles. Like interconnected vessels, risks from these private spheres inevitably impact workplace safety. Effective management must therefore prioritize understanding and influencing the behavioral risk patterns among specific demographics, for instance, younger HCWs. Additionally, it involves recognizing and responding to the significant variances across specific healthcare facilities over time, and implementing outbreak response mechanisms that are swift and hyper-local in their adaptation. As a foundation for such efforts, research must consistently integrate additional behavioral, institutional, and biological determinants of risk alongside those identified in this study.

## 5. Conclusions

HCWs constitute a distinctive sector of our society. The fluctuating nature of risk factors for infection highlights the need for adaptable preventive measures over time. Notably, the institutional subgroup emerged as the most influential variable in all risk factor analyses, emphasizing the importance of comprehending infection patterns within specific hospitals and departments as well as elderly and nursing homes. Furthermore, behavioral aspects are crucial for understanding the differences in infection rates. It is also important to remember that outbreaks can occur randomly as part of a stochastic process.

A higher seroprevalence in a specific institution might not necessarily indicate ineffective local infection control guidelines but reflect an earlier introduction of the virus into that institution by chance, causing subsequent local outbreak waves. Nevertheless, tailored standard operating procedures, specific to the institutional environment, can still make a significant difference by optimizing outbreak preparedness, early warning, and rapid response within the healthcare setting.

## Figures and Tables

**Table 1:** Description of the follow-up cohort included in the analyses with information before imputation. Potential breakthrough infections were identified by selecting participants who had received two or more vaccinations and a negative anti-N result at baseline but tested positive for anti-N antibodies at follow-up. Similarly, potential reinfections were characterized by participants exhibiting a positive anti-N result at baseline and an increased level of anti-N antibodies at follow-up.

Covariate	Category	Number of Participants *n* (%)	Qualitative Anti-N *n* (%)		Breakthrough Infection *n* (%)		Reinfection *n* (%)	
			Positive	Negative	Yes	No	Yes	No
Overall cohort		2351 (100.0%)	1036 (44.1%)	1315 (55.9%)	695 (38.8%)	1098 (61.2%)	84 (48.0%)	91 (52.0%)
Sex	Female	1740 (74.0%)	741 (42.6%)	999 (57.4%)	520 (37.3%)	874 (62.7%)	59 (48.8%)	62 (51.2%)
	Male	611 (26.0%)	295 (48.3%)	316 (51.7%)	175 (43.9%)	224 (56.1%)	25 (46.3%)	29 (53.7%)
Institutional subgroup	Barmherzige Brüder	141 (6.0%)	83 (58.9%)	58 (41.1%)	50 (46.3%)	58 (53.7%)	13 (39.4%)	20 (60.6%)
	Eichenau	22 (0.9%)	9 (40.9%)	13 (59.1%)	4 (23.5%)	13 (76.5%)	2 (40.0%)	3 (60.0%)
	Friedenheimer Brücke	34 (1.4%)	14 (41.2%)	20 (58.8%)	12 (37.5%)	20 (62.5%)	1 (100.0%)	0 (0.0%)
	General population	504 (21.4%)	232 (46.0%)	272 (54.0%)	50 (39.1%)	78 (60.9%)	17 (53.1%)	15 (46.9%)
	Medical Center of LMU	527 (22.4%)	200 (38.0%)	327 (62.0%)	175 (35.2%)	322 (64.8%)	8 (32.0%)	17 (68.0%)
	MK, Bogenhausen	193 (8.2%)	87 (45.1%)	106 (54.9%)	64 (38.6%)	102 (61.4%)	10 (55.6%)	8 (44.4%)
	MK, Harlaching	124 (5.3%)	58 (46.8%)	66 (53.2%)	46 (41.8%)	64 (58.2%)	4 (33.3%)	8 (66.7%)
	MK, Neuperlach	102 (4.3%)	34 (33.3%)	68 (66.7%)	30 (30.9%)	67 (69.1%)	3 (75.0%)	1 (25.0%)
	MK, Schwabing	248 (10.5%)	78 (31.5%)	170 (68.5%)	66 (28.6%)	165 (71.4%)	7 (58.3%)	5 (41.7%)
	MK, Thalkirchner St.	51 (2.2%)	20 (39.2%)	31 (60.8%)	16 (34.8%)	30 (65.2%)	1 (50.0%)	1 (50.0%)
	MS, Heilig Geist	32 (1.4%)	23 (71.9%)	9 (28.1%)	14 (60.9%)	9 (39.1%)	4 (66.7%)	2 (33.3%)
	MS, Rümannstraße	27 (1.1%)	12 (44.4%)	15 (55.6%)	10 (41.7%)	14 (58.3%)	0 (0.0%)	2 (100.0%)
	Obersendling	15 (0.6%)	8 (53.3%)	7 (46.7%)	7 (50.0%)	7 (50.0%)	0 (0.0%)	1 (100.0%)
	Seefeld	57 (2.4%)	22 (38.6%)	35 (61.4%)	18 (34.0%)	35 (66.0%)	3 (75.0%)	1 (25.0%)
	Tropical Institute	39 (1.7%)	20 (51.3%)	19 (48.7%)	16 (47.1%)	18 (52.9%)	1 (50.0%)	1 (50.0%)
	Vaccination center Riem	235 (10.0%)	136 (57.9%)	99 (42.1%)	117 (54.9%)	96 (45.1%)	10 (62.5%)	6 (37.5%)
Contact with patients	Yes	1211 (51.5%)	544 (44.9%)	667 (55.1%)	431 (39.9%)	648 (60.1%)	43 (44.3%)	54 (55.7%)
	No	762 (32.4%)	308 (40.4%)	454 (59.6%)	178 (35.2%)	328 (64.8%)	23 (51.1%)	22 (48.9%)
	Unknown *	378 (16.1%)	184 (48.7%)	194 (51.3%)	86 (41.3%)	122 (58.7%)	18 (54.5%)	15 (45.5%)
Smoking status	Never smoker	1636 (69.6%)	735 (44.9%)	901 (55.1%)	500 (40.0%)	750 (60.0%)	60 (50.0%)	60 (50.0%)
	Current smoker	343 (14.6%)	136 (39.7%)	207 (60.3%)	91 (34.1%)	176 (65.9%)	9 (36.0%)	16 (64.0%)
	Past smoker	367 (15.6%)	164 (44.7%)	203 (55.3%)	103 (38.0%)	168 (62.0%)	15 (50.0%)	15 (50.0%)
	Unknown *	5 (0.2%)	1 (20.0%)	4 (80.0%)	1 (20.0%)	4 (80.0%)	-	-
Vaccination scheme	No vaccination **	242 (10.3%)	119 (49.2%)	123 (50.8%)	-	-	13 (54.2%)	11 (45.8%)
	One vaccination	226 (9.6%)	139 (61.5%)	87 (38.5%)	-	-	33 (54.1%)	28 (45.9%)
	Two vaccinations	1779 (75.7%)	744 (41.8%)	1035 (58.2%)	665 (39.3%)	1028 (60.7%)	36 (41.9%)	50 (58.1%)
	Three vaccinations	104 (4.4%)	34 (32.7%)	70 (67.3%)	30 (30.0%)	70 (70.0%)	2 (50.0%)	2 (50.0%)
Household size	One person	667 (28.4%)	262 (39.3%)	405 (60.7%)	163 (33.3%)	327 (66.7%)	23 (43.4%)	30 (56.6%)
	2 people	803 (34.2%)	333 (41.5%)	470 (58.5%)	219 (35.9%)	391 (64.1%)	27 (47.4%)	30 (52.6%)
	3 people	367 (15.6%)	169 (46.0%)	198 (54.0%)	121 (41.3%)	172 (58.7%)	12 (50.0%)	12 (50.0%)
	4 people	349 (14.8%)	176 (50.4%)	173 (49.6%)	119 (44.1%)	151 (55.9%)	15 (50.0%)	15 (50.0%)
	5+ people	119 (5.1%)	73 (61.3%)	46 (38.7%)	54 (58.1%)	39 (41.9%)	5 (62.5%)	3 (37.5%)
	Unknown *	46 (2.0%)	23 (50.0%)	23 (50.0%)	19 (51.4%)	18 (48.6%)	2 (66.7%)	1 (33.3%)
Intake of immunosupp. drugs	Yes	83 (3.5%)	34 (41.0%)	49 (59.0%)	20 (31.7%)	43 (68.3%)	2 (40.0%)	3 (60.0%)
	No	2252 (95.8%)	996 (44.2%)	1256 (55.8%)	672 (39.0%)	1049 (61.0%)	81 (48.2%)	87 (51.8%)
	Unknown *	16 (0.7%)	6 (37.5%)	10 (62.5%)	3 (33.3%)	6 (66.7%)	1 (50.0%)	1 (50.0%)

* The values for the “unknown” category of the corresponding variables have been imputed for the modeling process; ** These participants were vaccinated on the day of baseline blood sampling and thus considered as “not vaccinated”.

## Data Availability

Data are subject to data protection regulations and can be made available upon reasonable request to the corresponding author. To facilitate reproducibility and reuse, the code used to perform the analyses and generate the figures was made available in an open-source GitHub repository (https://gitlab.lrz.de/TROP.noemi.castelletti/kocoimpf_followup) (accessed on 22 July 2024).

## Supplementary Materials

The following supporting information can be downloaded at: https://www.mdpi.com/article/10.3390/v16101556/s1, Figure S1: Risk factor analysis for only Omicron-related infections (*n* = 2176, Model 2). A person with a prior infection was identified as being anti-N positive only at follow-up. Individuals who were anti-N positive at baseline were excluded. Findings are derived from multiple imputations. The obtained value of the model evaluation unison pooled AUC was 0.6400. (A) Estimates for categorical variables. (B) Estimates for continuous variables with 95% CI represented by the gray shaded region. Figure S2: Risk factor analysis for reinfections (*n* = 175, Model 4). A person with a prior infection was identified as being anti-N positive at follow-up. Only individuals who were anti-N positive at baseline were included. Findings are derived from multiple imputations. The obtained value of the model evaluation unison pooled AUC was 0.6803. (A) Estimates for categorical variables. (B) Estimates for continuous variables with 95% CI represented by the gray shaded region.
