# Supplementary material for: Understanding the Omicron Variant Impact in Healthcare Workers: Insights from the Prospective COVID-19 Post-Immunization Serological Cohort in Munich (KoCo-Impf) on Risk Factors for Breakthrough and Reinfections
Source: Viruses. 2024 Sep 30;16(10):1556. doi: 10.3390/v16101556 (PMC11512372; doi:10.3390/v16101556)
Supplement: Supplementary file 1 [file viruses-16-01556-s001.zip › viruses-3152129-supplementary.pdf]

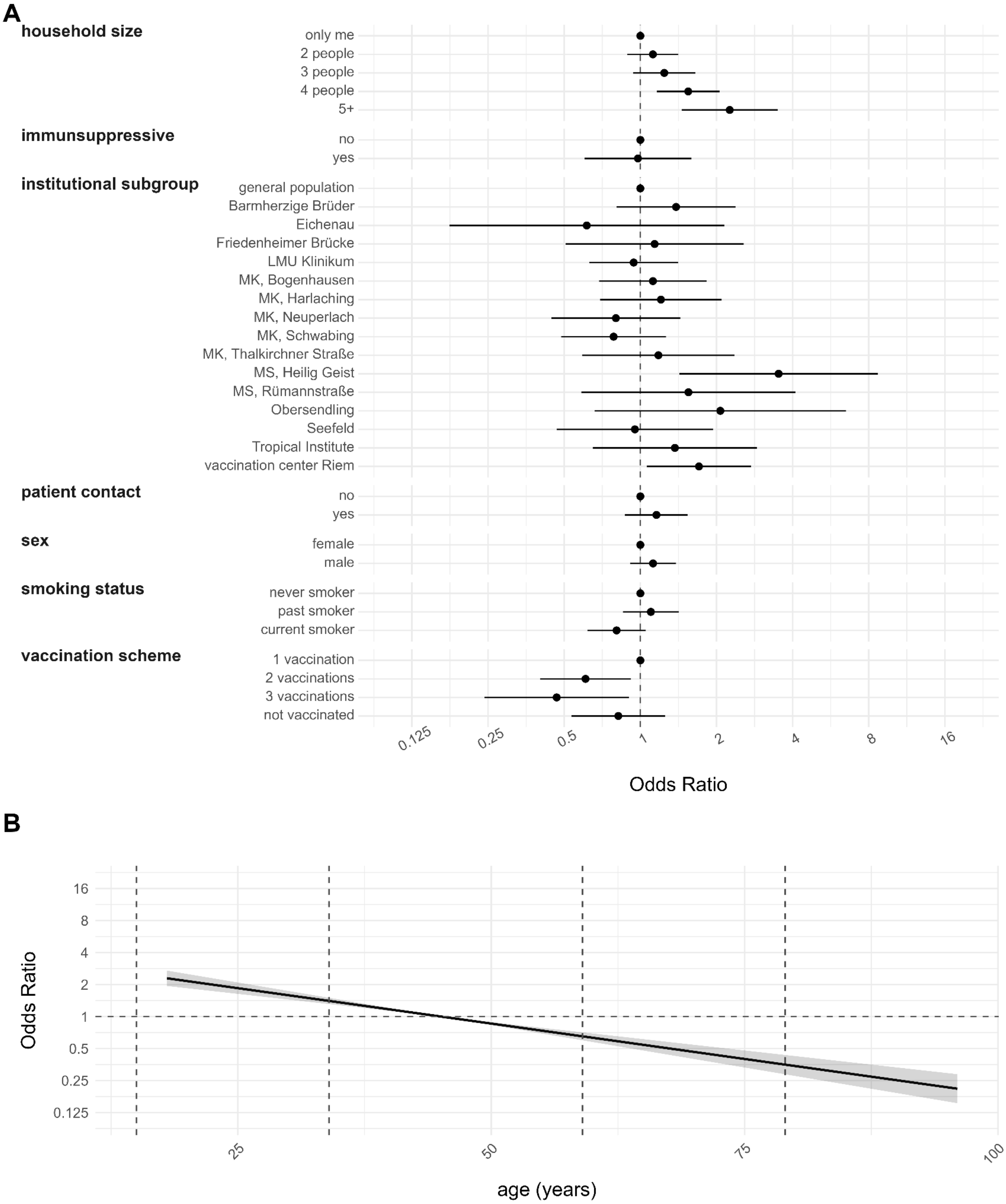

Figure S1. Risk factor analysis for only Omicron-related infections (n = 2176, Model 2). A person with a prior infection was identified as being anti-N positive only at follow-up. Individuals who were anti-N positive at baseline were excluded. Findings are derived from multiple imputations. The obtained value of the model evaluation unison pooled AUC was 0.6400. (**A**) Estimates for categorical variables. (**B**) Estimates for continuous variables with 95% CI represented by the gray shaded region.

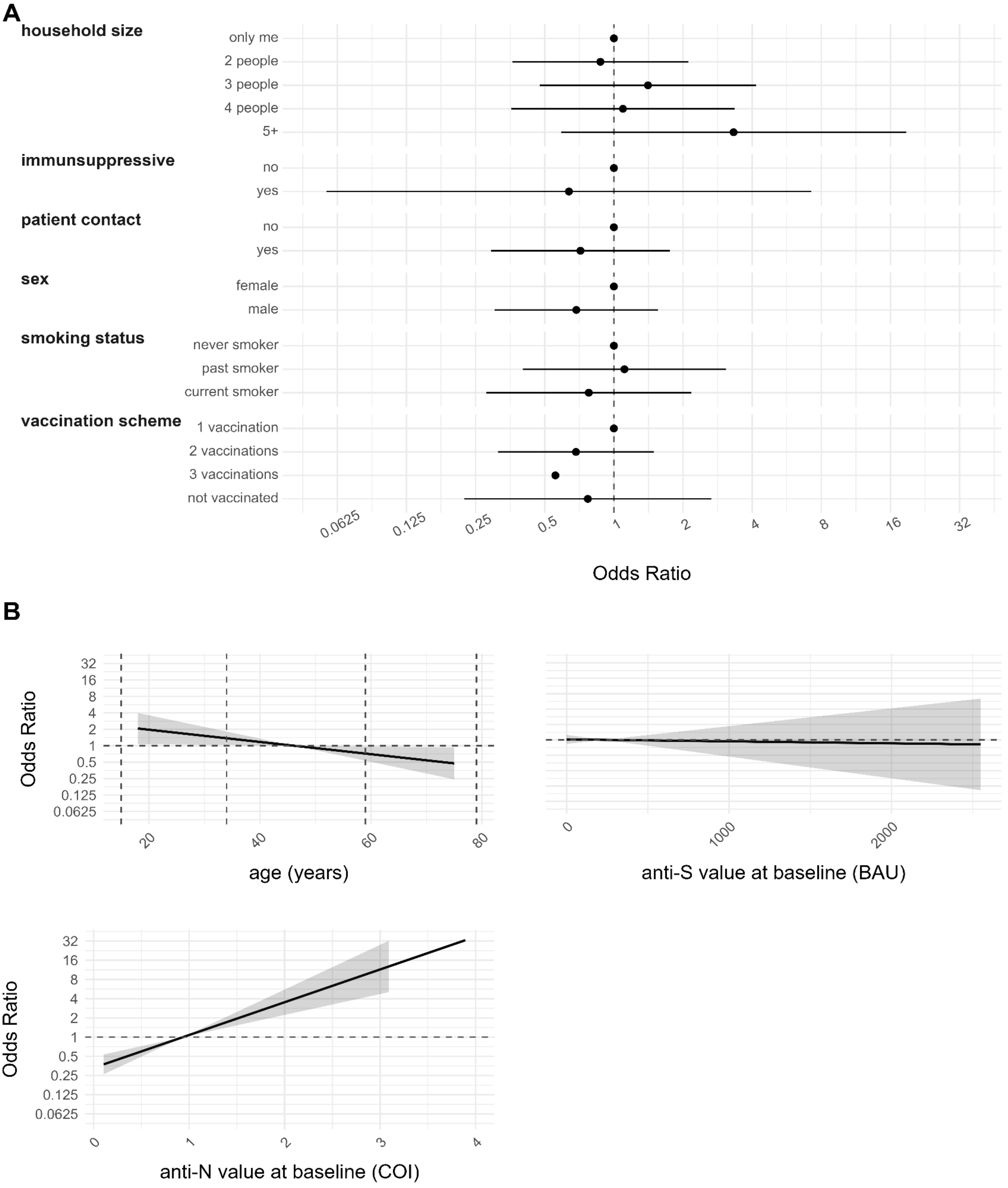

Figure S2. Risk factor analysis for reinfections (n = 175, Model 4). A person with a prior infection was identified as being anti-N positive at follow-up. Only individuals who were anti-N positive at baseline were included. Findings are derived from multiple imputations. The obtained value of the model evaluation unison pooled AUC was 0.6803. (**A**) Estimates for categorical variables. (**B**) Estimates for continuous variables with 95% CI represented by the gray shaded region.
